# Labour Analgesia When Epidural Is Not a Choice: Tramadol versus Pentazocine

**DOI:** 10.1155/2014/930349

**Published:** 2014-04-07

**Authors:** Jyothi Shetty, Ashwini Vishalakshi, Deeksha Pandey

**Affiliations:** Department of Obstetrics and Gynecology, Manipal University, Manipal 576104, Karnataka, India

## Abstract

*Background*. Parenteral opioids, thus, are still popular for pain relief in labor in many countries throughout the world. *Aim*. To evaluate and compare the efficacy of intramuscular tramadol and pentazocine in the first stage of labor. *Method.* Sixty-five patients were divided into pentazocine group and tramadol group. Subjects received either 30 mg pentazocine or 1 mg/kg tramadol intramuscularly. Pain was assessed using visual analog scale (VAS) before the administration of the drug, at 1 h, 2 h, 4 h, and at full dilatation. Maternal and neonatal side effects were determined. *Results*. Analgesic effect of the two drugs was not significantly different. Neither of these analgesics was effective towards the end of the first stage. However, in the tramadol group, the majority of women (55%) rated pain as severe, whereas in the pentazocine group, the majority of women (60%) rated pain as moderately severe. There were not many side effects with either of the drug in the given dosage. Mean injection to delivery interval was significantly shorter in the tramadol group as compared to the pentazocine group. *Conclusion.* Pentazocine or tramadol can be given for labor pain relief as an alternative to epidural analgesia in resource poor setting.

## 1. Introduction

Pain management during labour is an essential part of good obstetric care. Though this severe pain during labour is not life threatening, it can have neuropsychological consequences. Postnatal depression may be more common when labour analgesia is not used [1]. Pain during labor has also been correlated with the development of posttraumatic stress disorder [2].

It has been proven beyond a doubt that epidural analgesia, when compared with other methods, provides superior analgesia for labor. However there can be situations where either it is not available or it is not feasible. Parenteral opioids, thus, are still popular for pain relief in labor in many countries throughout the world.

A recent Cochrane review emphasized that as parenteral opioid drugs are very widely used for labor analgesia, it is important that more research is carried out so that women can make informed choices about these forms of pain relief [3].

Use of parenteral opioids for labour pain relief has been quite common, pethidine being the most commonly used drug in this group worldwide [4]. Studies on pethidine have, however, raised concerns about its adverse effects on the parturient and the newborn. It has been implicated to result in neonatal behavioral and feeding problems up to six weeks after delivery [5]. These concerns about pethidine have promoted a search for alternative opioids like pentazocine and tramadol.

Tramadol is a synthetic analogue of codeine and is a centrally acting agent. It has a relatively low affinity for opiate receptors. Studies have shown that tramadol is an effective analgesic without the maternal and neonatal respiratory depression common to other opioids and it does not delay gastric emptying. Pentazocine, a benzomorphan derivative, is a synthetic opioid with both agonist and weak antagonistic action. It produces analgesia with little or no respiratory depression. When used for the relief of labor pain many studies have demonstrated equal analgesic effect with a better safety profile compared with pethidine.

These two may be useful in producing effective pain relief in labor without the adverse effects seen in pethidine. Tramadol or pentazocine can be used as labor analgesics with minimum cost and less training as compared to the proven epidural analgesia that requires trained staff and equipment and has higher cost. It also avoids the side effects associated with epidural analgesia like hypotension, foetal heart rate changes, impaired motor ability, shivering, urinary retention, delayed pushing, and a prolonged second stage of labour.

In this study we aimed to evaluate and compare the efficacy of intramuscular tramadol and pentazocine in the first stage of labour.

## 2. Material and Methods

This prospective study was carried out in the labour ward at a tertiary care centre for one year. The study protocol was approved by the institutional ethics committee. All enrolled women provided written informed consent for participation. A total of 65 women with normal singleton, cephalic-presenting foetuses, between 37- and 41-week period of gestation, and who requested analgesia in labour were recruited for the study. Only those women who were having at least 2 regular contractions every 10 min with cervical dilatation of at least 3 cm were included. Patients with previous uterine scar, multiple gestation, malpresentation, absent membranes, antepartum haemorrhage, cephalopelvic disproportion, and pregnancies complicated with medical complications were excluded.

A total of 80 women, 40 in each arm were recruited using computer generated random number table, as a convenient sample. Written consent was taken from these women once they crossed 37 weeks of gestation (i.e., once they reached term). All these women were informed about the study; however they were blinded as to which arm of study they belonged to. Among the women recruited for the pentazocine group nine women went for caesarean before the drug could be administered (five for nonreassuring labour admission test and four for failed induction), four women refused to participate in the study, and two within one hour of administration of pentazocine requested epidural analgesia. Thus, unfortunately, 15 women had to be excluded from the final analysis.

The “pentazocine group” received 30 mg pentazocine (Ranbaxy) and the “tramadol group” received tramadol (Zydac Aliloc) 1 mg/ kg intramuscularly at request for labour analgesia. Clinical data and analgesic efficacy were assessed. Pain was assessed by a 10 cm long visual analogue scale with 0 representing no pain and 10 as the worst pain. Pain was then graded into mild (scores of 0–3), moderate (scores of 4–6), and severe (scores of 7–10). Pain was assessed before the administration of the drug (0 h), at 1 h, 2 h, and 4 h following drug administration, and at full dilatation. Simultaneously maternal pulse rate, blood pressure, respiratory rate, and foetal heart rate were recorded before administering the drug (0 h), at 1 h, 2 h, and 4 h, and at full dilatation. Side effects like sedation, vomiting, drowsiness, tachycardia, and foetal distress were noted following the administration of the drug. Maternal sedation was assessed on a three-point scale as 0 = alert, 1 = drowsy, and 2 = asleep. Intrapartum monitoring was done according to the standard labour ward protocol using the partogram. The time interval between drug administration and delivery was recorded. Neonatal evaluation was done by the neonatologist using APGAR score. The neonatologist was informed about the type of analgesia given to the mother. Naloxone usage for any presumed opioid induced respiratory depression was recorded.

### 2.1. Statistical Analysis

Statistical analysis of the data was done using SPSS 11.0. Results were expressed as mean ± standard deviation (SD). Qualitative analysis was done using Student's *t*-test. For quantitative analysis Chi-square test was used. Nonparametric data were compared with Mann-Whitney *U* test. A *P* value of <0.05 was considered significant.

## 3. Results 

Of the total 65 patients who requested labour analgesia, 40 women were in the tramadol group and 25 in the pentazocine group. All of the patients in this study received only a single dose of either tramadol or pentazocine.

Maternal characteristics like age, height, weight, blood pressure, parity, gestational age, and cervical dilatation at initiation of analgesia were comparable (Table 1).

Median pain score before administering the drug (at 0 h) was 8 in both tramadol and pentazocine groups. This score decreased to a median pain score of 7 in the tramadol group and 6 in the pentazocine group one hour after drug administration. Difference in pain relief between the two drugs was not significant (*P* > 0.05). Twenty-three women in the tramadol group and three in the pentazocine group delivered within four hours of the analgesic administration. In the patients who had not delivered the median pain score was 8 at 4 h in both groups. Thus, the analgesic effect of tramadol and pentazocine was comparable. At the end of the first stage the median pain score was 10 in both groups (Table 2).

**Figure 1:**
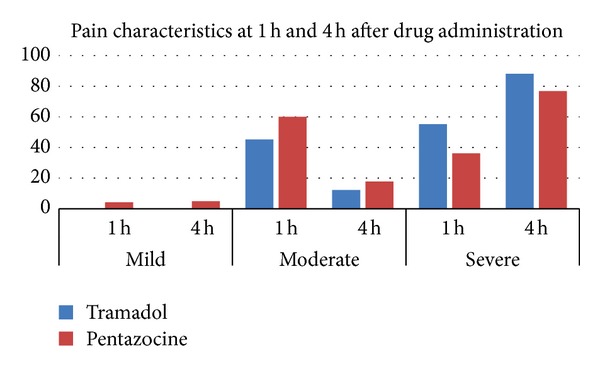
Pain characteristics at 1 h and 4 h after drug administration. *Pain perception at the end of 4 h was severe in 88.2% in the tramadol group while 77.2% perceived it as severe in the pentazocine group. This difference was statistically significant (*P* = 0.017).

On characterizing pain into mild, moderate, and severe, at 1 h after drug administration mild pain was experienced by one (4%) patient in the pentazocine group and none in the tramadol group. In the tramadol group, the majority of women (55%) rated pain as severe, whereas in the pentazocine group, the majority of women (60%) rated pain as moderately severe. However this difference was not statistically significant. At 4 h after drug administration mild pain was experienced by only one (4.5%) patient in the pentazocine group. The majority of women in both groups rated pain as severe. However only 88.2% women in the tramadol group experienced severe pain compared to 77.2% in the pentazocine group and this difference was statistically significant (*P* < 0.05) (Figure 1).

The mode of delivery did not differ statistically between the two groups. Birth weight was also found to be comparable (2.86 ± 0.41 kg in the tramadol group and 2.94 ± 0.365 kg in the pentazocine group). Mean injection to delivery interval was shorter in the tramadol group as compared to the pentazocine group and the difference was statistically significant. Mean Apgar score of the neonates at 1 and 5 min in both groups were also comparable (Table 3).

The side effect profile of these two opioids was found to be not significant in our study. Only one lady had nausea and vomiting, while one felt drowsy. Both these women belonged to the pentazocine group.

## 4. Discussion

In our study we found that the analgesic effect of the two drugs was not significantly different. Neither of these analgesics was effective towards the end of the first stage. However in the tramadol group, the majority of women (55%) rated pain as severe, whereas in the pentazocine group, the majority of women (60%) rated pain as moderately severe. There were not many side effects with either of the drugs in the given dosage.

We also noticed that mean injection to delivery interval was shorter in the tramadol group as compared to the pentazocine group and the difference was statistically significant. Similarly, another study from India also noted that injection to delivery interval was significantly shorter in the tramadol group [6]. The same results were obtained from one more recent study wherein the investigators found that tramadol significantly shortens the duration of labour [7].

A randomized controlled trial done in Nigeria also showed results similar to us. They concluded that both pentazocine and tramadol are safe for the relief of labor pain.

Pentazocine, however, provides better pain relief than tramadol in labor, but it is associated with higher incidence of drowsiness. There was no significant difference between the two drugs in outcome of labor and neonatal side effects [8].

Jain et al. compared intramuscular opioids with epidural analgesia in labour and concluded that, in developing nations where availability of facilities is the main limiting factor, intramuscular opioids can be considered suitable alternatives [9].

The limitations of this study are that opioids were administered only once in this study, the effect of which lasts for approximately 4 hours. Since the duration of labour is usually longer, the analgesic effect does not last long enough to provide adequate analgesia for the entire duration of labour. The sample size is small, so it is difficult to extrapolate these results into the general population.

The positive point in our study methodology was that none of the other studies comparing tramadol and pentazocine have used VAS for assessing the analgesic effect of these drugs. VAS is a measurement instrument used for the quantification of subjective characteristics that cannot be directly measured like pain perception. It has been suggested that this scaling system has superior metrical characteristics as compared to any other classification of pain (like mild, moderate, and severe). The other important finding was that all the previous studies have used 100 mg of tramadol, whereas we tried to tailor it with 1 mg/kg body weight and got similar efficacy. This concept might help reduce the adverse effect of the drug while still maintaining the desired analgesic effect.

## 5. Conclusion

Tramadol and pentazocine both offer similar pain relief during labor and are safe for the mother and the baby. The analgesic effect of pentazocine was sustained up till 4 h as compared to tramadol where the analgesic effect wears off early. However tramadol seems to shorten the overall duration of labour. Pentazocine or tramadol can be given for labour pain relief as an alternative to epidural analgesia in resource poor setting. Further studies with multidose regimen of these analgesics are worth looking into.

## Figures and Tables

**Table 1:** Maternal characteristics among the two groups.

Characteristics	Tramadol *n* = 40	Pentazocine n = 25	P value
Age (mean ± SD)	27.15 ± 2.99	26.68 ± 3.23	0.552
Height cm (mean ± SD)	155.63 ± 6.50	155.04 ± 5.35	0.708
Weight kg (mean ± SD)	64.93 ± 6.71	64.80 ± 6.55	0.941
Primigravida (%) Multigravida	25 (62.5) 15 (37.5)	19 (76) 06 (24)	0.326
Gestational age weeks (mean ± SD)	37.73 ± 1.62	38.24 ± 1.36	0.190
Cervical dilatation at initiation of analgesia cm (mean ± SD)	3.11 ± 0.737	3.12 ± 0.726	0.130
Systolic BP before analgesia mm Hg (mean ± SD)	123.2 ± 6.12	123.2 ± 7.21	1.000

**Table 2:** Comparison of pain score (VAS) among the two groups.

	Tramadol			Pentazocine			*P* value
		Median	Range		Median	Range	
Before injection (0 h)	*n* = 40	8	4–10	*n* = 25	8	5–10	0.281
1 hour	*n* = 40	7	4–10	*n* = 25	7	2–10	0.073
4 hours	*n* = 17	8	5–10	*n* = 22	8	2–10	0.732
End of first stage	*n* = 36	10	6–10	*n* = 25	10	6–10	0.914

**Table 3:** Labour characteristics and neonatal outcome among the two groups.

Labour	Tramadol *n* = 40 (%)	Pentazocine *n* = 25 (%)	*P* value
Induced	22 (55)	8 (32)	0.388
Normal vaginal delivery	26 (65)	23 (92)	0.080
Forceps delivery	5 (12.5)	1 (4)	0.080
Vacuum delivery	3 (7.5)	1 (4)	0.080
Caesarean section	6 (15)	0	
Birth weight in kg (mean ± SD)	2.86 ± 0.41	2.94 ± 0.365	0.428
Mean APGAR (1 min)	8.93	9.00
Mean APGAR (5 min)	9.98	10.00
Injection delivery interval in min (mean ± SD)	265.91 ± 103.35	430.28 ± 117.29	0.0001*

(*P value < 0.05; ie: statistically significant).

## References

[1] Hiltunen P, Raudaskoski T, Ebeling H, Moilanen I (2004). Does pain relief during delivery decrease the risk of postnatal depression?. *Acta Obstetricia et Gynecologica Scandinavica*.

[2] Soet JE, Brack GA, Dilorio C (2003). Prevalence and predictors of women’s experience of psychological trauma during childbirth. *Birth*.

[3] Ullman R, Smith LA, Burns E, Mori R, Dowswell T (2010). Parenteral opioids for maternal pain relief in labour. *Cochrane Database of Systematic Reviews*.

[4] Bricker L, Lavender T (2002). Parenteral opioids for labor pain relief: a systematic review. *American Journal of Obstetrics and Gynecology*.

[5] Nissen E, Widström A-M, Lilja G (1997). Effects of routinely given pethidine during labour on infants’ developing breastfeeding behaviour. Effects of dose-delivery time interval and various concentrations of pethidine/norpethidine in cord plasma. *Acta Paediatrica*.

[6] Tripti N, Jyotsna A (2006). Pain relief in labor-tramadol versus pentazocine. *The Journal of Obstetrics and Gynecology of India*.

[7] Chandnani K, Sainee HB (2013). Pain relief in labour: tramadol versus pentazocine. *International Journal of Reproduction, Contraception, Obstetrics and Gynecology*.

[8] Kuti O, Faponle AF, Adeyemi AB, Owolabi AT (2008). Pain relief in labour: a randomized controlled trial comparing pentazocine with tramadol. *Nepal Journal of Obstetrics & Gynaecology*.

[9] Jain S, Arya VK, Gopalan S, Jain V (2003). Analgesic efficacy of intramuscular opioids versus epidural analgesia in labor. *International Journal of Gynecology and Obstetrics*.

